# Bottled and Well Water Quality in a Small Central Appalachian Community: Household-Level Analysis of Enteric Pathogens, Inorganic Chemicals, and Health Outcomes in Rural Southwest Virginia

**DOI:** 10.3390/ijerph19148610

**Published:** 2022-07-15

**Authors:** Alasdair Cohen, Md Rasheduzzaman, Amanda Darling, Leigh-Anne Krometis, Marc Edwards, Teresa Brown, Tahmina Ahmed, Erin Wettstone, Suporn Pholwat, Mami Taniuchi, Elizabeth T. Rogawski McQuade

**Affiliations:** 1 Department of Population Health Sciences, Virginia Tech, Blacksburg, VA 24061, USA; mdrashed@vt.edu (M.R.); amandavd@vt.edu (A.D.); 2 Department of Civil & Environmental Engineering, Virginia Tech, Blacksburg, VA 24061, USA; edwardsm@vt.edu; 3 Department of Biological Systems Engineering, Virginia Tech, Blacksburg, VA 24060, USA; lehenry@vt.edu; 4 Department of Natural Sciences, University of Virginia’s College at Wise, Wise, VA 24293, USA; tlb9fd@uvawise.edu; 5 Department of Engineering Systems and Environment, University of Virginia, Charlottesville, VA 22908, USA; ta6n@virginia.edu (T.A.); mt2f@virginia.edu (M.T.); 6 Division of Infectious Diseases and International Health, University of Virginia, Charlottesville, VA 22908, USA; egw6vx@virginia.edu (E.W.); sp4vs@virginia.edu (S.P.); elizabeth.rogawski.mcquade@emory.edu (E.T.R.M.); 7 Department of Biomedical Engineering, University of Virginia, Charlottesville, VA 22908, USA; 8 Department of Epidemiology, Emory University, Atlanta, GA 30322, USA

**Keywords:** drinking water, environmental health, enteric pathogens, metals, well water, bottled water, rural health, Virginia, Appalachia

## Abstract

Consumption of unsafe drinking water is associated with a substantial burden of disease globally. In the US, ~1.8 million people in rural areas lack reliable access to safe drinking water. Our objective was to characterize and assess household-level water sources, water quality, and associated health outcomes in Central Appalachia. We collected survey data and water samples (tap, source, and bottled water) from consenting households in a small rural community without utility-supplied water in southwest Virginia. Water samples were analyzed for physicochemical parameters, total coliforms, *E. coli*, nitrate, sulfate, metals (e.g., arsenic, cadmium, lead), and 30+ enteric pathogens. Among the 69% (*n* = 9) of households that participated, all had piped well water, though 67% (*n* = 6) used bottled water as their primary drinking water source. Total coliforms were detected in water samples from 44.4% (*n* = 4) of homes, *E. coli* in one home, and enteric pathogens (*Aeromonas*, *Campylobacter*, *Enterobacter*) in 33% (*n* = 3) of homes. Tap water samples from 11% (*n* = 1) of homes exceeded the EPA MCL for nitrate, and 33% (*n* = 3) exceeded the EPA SMCL for iron. Among the 19 individuals residing in study households, reported diarrhea was 25% more likely in homes with measured *E. coli* and/or specific pathogens (risk ratio = 1.25, cluster-robust standard error = 1.64, *p* = 0.865). Although our sample size was small, our findings suggest that a considerable number of lower-income residents without utility-supplied water in rural areas of southwest Virginia may be exposed to microbiological and/or chemical contaminants in their water, and many, if not most, rely on bottled water as their primary source of drinking water.

## 1. Introduction

Access to safe and affordable drinking water is necessary for human health, development, and dignity. Over the last few decades there have been significant gains in the water, sanitation, and hygiene sector globally. However, an estimated two billion people, mostly in low- and middle-income countries (LMICs), still lack access to safely managed drinking water; globally, disparities in access to safe water are most severe in rural areas [1,2]. Consumption of unsafe drinking water is associated with a substantial burden of disease [3,4,5]. Of the ~1.4 million diarrhea-attributed deaths in 2016, inadequate safe water access accounted for ~0.5 million [5]. A number of cancers (e.g., kidney, urinary, bladder) are known to be associated with chronic exposure to heavy metals in drinking water, as are other adverse health outcomes such as hypertension, cardiovascular disease, and impaired cognitive development [6,7,8,9].

Unbeknown to many, ~1.8 million people living in rural areas of the US lack reliable access to safe drinking water (estimate derived from data in WHO/UNICEF report, annex three) [1]. A recent nationwide analysis of US Census and US Environmental Protection Agency (EPA) Safe Drinking Water Act (SDWA) data identified Appalachia as one of the US regions with the highest rates of water utility violations and numbers of households without complete plumbing [10]. Appalachia, a predominantly rural region in the eastern US, is home to ~26 million people living in 423 counties across parts of the US States of Alabama, Georgia, Kentucky, Maryland, Mississippi, New York, North Carolina, Ohio, Pennsylvania, South Carolina, Tennessee, and Virginia, as well as all the counties in the State of West Virginia [11].

In low-income rural areas that lack access to safe, utility-provided, drinking water, many households rely on private well water, bottled water, and roadside springs. Yet, our understanding of which rural Appalachian regions, communities, and populations have higher risks of exposure to contaminated drinking water is severely limited by a lack of data, and we know even less about associated impacts on health [12,13,14,15]. For example, although as many as 12 million Americans are estimated to suffer from neglected parasitic infections [16,17], a recent (382 page) report on “Health Disparities in Appalachia” did not discuss drinking water, sanitation, or enteric diseases [18]. Recruiting participants from at-risk populations in rural Appalachia and other rural regions of the US is often challenging [19,20,21], which may partially explain both the relative lack of available data in this domain, and why many published water and health-focused studies from rural Appalachia are based on relatively small sample sizes.

Recent studies have identified water-related disparities in Central Appalachia, found microbiological and heavy metal contamination in Central Appalachian roadside springs commonly used to meet household potable water needs, and suggest that rates of rural bottled water use may be increasing in some areas of the region [12,22,23]. US Census Bureau estimates also indicate that in Virginia’s most southwestern counties of Lee, Wise, and Scott, ~10% of homes in this region of Central Appalachia lack complete plumbing or hot and cold running water [24]. In addition, the Appalachian Regional Commission classifies both Lee and Wise counties as “distressed”, a designation applied to the “most economically depressed counties” in Appalachia [25,26].

Given the relative lack of household-level data in this area, our objective for this study was to elucidate potential exposures to microbiological and chemical contaminants in drinking water by characterizing and quantifying the use and quality of household-level drinking water sources, as well as associated health outcomes and socioeconomic factors, in lower-income households without utility-supplied water in a rural community in Wise County, Virginia.

## 2. Materials & Methods

### 2.1. Study Setting

With support from the Wise County Public Service Authority (PSA) in Virginia, in November and December of 2021, researchers from Virginia Tech visited households in a small community in a rural area of Wise County. Households were eligible for inclusion in the study if they were located within the community under consideration for a PSA water supply extension project at that time. The community was situated in a narrow valley, underlain by alluvium (sandstone and shale) and coal mine spoil material, with a relatively shallow (~3 m) depth to the water table and subject to frequent flooding [27].

### 2.2. Data Collection

We used a cross-sectional design and administered face-to-face interviews using a structured survey that included items from a standardized PSA needs assessment as well as questions about water sources, preferences, related perceptions and behaviors, and individual-level reported health outcomes. In addition to survey items previously used in other low-income rural settings [28,29,30], additional survey questions were developed in consultation with the PSA.

For households that consented to water sampling and testing, we collected multiple samples from two to three sources per home: tap water, source water (tap after sterilizing the faucet with 70% isopropyl alcohol solution and flushing/running the water for five minutes), and bottled water or roadside spring water (if used as a primary drinking water source). Autoclaved sampling bottles were pre-labeled with household-linked code numbers which were also used for labeling surveys.

Physicochemical parameters were measured immediately after each visit to a household using a YSI Multiparameter Water Quality Meter (YSI Inc., Yellow Springs, OH, USA) to record duplicate measurements for pH, temperature, conductivity (μS/cm), and dissolved oxygen (%DO). Sampling bottles for additional analyses were transported on ice and analyzed or processed within ~8 h of collection.

### 2.3. Water Sample Analyses

Duplicate samples were tested for *E. coli*, an indicator of fecal contamination, and total coliforms, via IDEXX Colilert Defined Substrate and IDEXX Quanti-Tray/2000 (IDEXX, Westbrook, MN, USA) at UVA-Wise and Virginia Tech (Standard Method 9223) [31]. Samples were also tested for nitrate (NO_3_^−^) and sulfate (SO_4_^2−^) using a Hach DR850 portable colorimeter (Hach Company, Loveland, CO, USA). Additionally, samples were processed with 2% trace metal grade nitric acid by volume and then analyzed for metals including arsenic, cadmium, chromium, copper, iron, lead, manganese, and silver using a Thermo Electron iCAP-RQ ICP-MS at Virginia Tech (Standard Methods 3030D, 3125B) [31].

Considering the importance of identifying specific pathogenic organisms [32], source water from each household was filtered and concentrated using 0.2 μm concentrating pipettes (CP) for 1 L, and 0.05 μm CPs for 500 mL, with an InnovaPrep Concentrating Pipette Select, and then eluted in 0.075% Tween 20/25 mM Tris wet foam elution buffer (InnovaPrep LLC, Drexel, MO, USA). Elutions were shipped on dry ice from Virginia Tech to the University of Virginia for extraction and analysis using a custom-designed TaqMan array card, high throughput RT-qPCR assay, to detect 30+ viral, bacterial, protozoal, and helminthic pathogens [33,34,35].

### 2.4. Data Sharing, Ethics, & Statistical Analyses

Prior to initiating data collection, we uploaded pre-specified study protocols to the Open Science Framework [36]. Within six weeks of data collection, summary sheets with water quality results for each participating household, with reference to EPA SDWA standards, were provided to the PSA (by A.C.); the PSA then matched study codes to addresses and provided participants their water results.

Although neither bottled nor private well water are regulated by the SDWA, we contextualized measured water quality results via comparison with EPA health- and aesthetic-based standards. Because US EPA maximum contaminant levels (MCL) and secondary MCL (SMCL) are established based on health risk data as well as considerations related to treatment methods and costs, we report our results in terms of concentrations exceeding both full and half EPA MCLs and SMCLs, as appropriate.

Our study was approved by Virginia Tech’s Institutional Review Board (VT-IRB #21-763) and this manuscript was prepared in accordance with STROBE reporting guidelines [37]. Statistical analyses (two-sided tests, standard *p* < 0.05 threshold for significance, and no imputation for missing data) and modeling were conducted (by A.C.) using Stata (Stata/MP v16.1, StataCorp, College Station, TX, USA) and then replicated (by M.R.) using R (v4.1.1).

## 3. Results

All 15 homes in the cluster/community were eligible for inclusion in our study; however, 27% (*n* = 4) declined to support the utility’s proposed water extension proposal, and three of these four households also declined to participate in our study. We were unable to contact a respondent in one occupied home (after multiple attempts), and two homes were vacant when we initiated the study. Thus, of the non-vacant homes in the community at the time of our visits (*n* = 13), 69% (*n* = 9) agreed to participate in our study.

### 3.1. Household Characteristics

All nine of the eligible and non-vacant households that agreed to participate in our study had working access to piped private well water in their homes, though 67% (*n* = 6) used bottled water as their primary drinking water source. None of the 33% (*n* = 3) of households using well water as their primary drinking water source reported treating their water (any method). Reported annual household incomes ranged from <$33,000 to no more than $48,000, with 67% (*n* = 6) of households reporting incomes of <$43,000/year (Table 1).

Reported incomes were higher, overall, in homes using bottled water compared with those using well water, though the difference was not statistically significantly (Wilcoxon rank-sum test, *p* = 0.231). Heads of household were younger, on average, in homes using bottled water (mean = 51.0, *n* = 6) compared with those using private well water (mean = 66.3, *n* = 3), though the difference was also not statistically significant (*t*-test with unequal variance, *p* = 0.104).

### 3.2. Water Quality Results

We detected total coliforms in water samples from 44.4% (*n* = 4) of households, including one bottled water sample. *E. coli* (an indicator of fecal contamination) was detected in tap and source water samples from one home; although the source water sample concentration (mean of duplicate samples) was low (1.5 MPN/100 mL, standard deviation [SD] = 3.0 MPN/100 mL), it still exceeded the US EPA MCL for *E. coli* [38]. We also detected bacterial pathogens (using an RT-qPCR cutoff of Ct < 35) in source water samples from 33.3% (*n* = 3) of homes (including the home with *E. coli*), with detection of *Campylobacter* and *Enterobacter* in 22.2% (*n* = 3) of homes, and *Aeromonas* in 11.1% (*n* = 1); no other pathogens were detected at a Ct < 35 threshold (Table 2).

**Figure 1:**
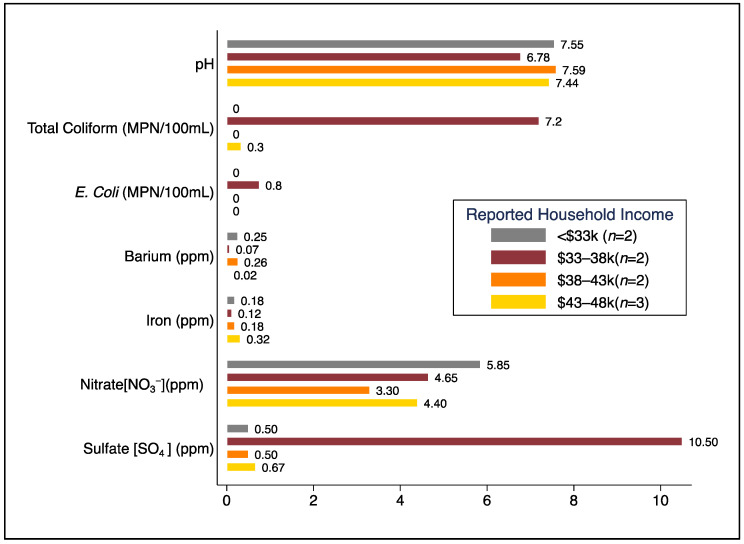
Comparison of mean concentrations for selected water quality parameters from source water samples (tap after five-minute flush) by reported household income brackets.

With regard to non-microbiological water quality markers with US EPA MCL and SMCL regulatory standards, tap water samples from one home exceeded the EPA MCL for nitrate (10 ppm), and tap water samples from 33.3% (*n* = 3) of homes exceeded the SMCL for iron (0.3 ppm) [38,39]. Tap water samples from 44.4% (*n* = 4) of homes exceeded half of the MCL for nitrate, and 55.5% (*n* = 5) exceeded half of the SMCL for iron. Half of the bottled water samples we tested (50%, *n* = 3) exceeded half of the US EPA MCL for nitrate. We did not observe differential trends for these water quality parameters by levels of reported household income, though our observations were limited to only 2–3 households per income bracket (Figure 1).

### 3.3. Health Outcomes

Diarrhea (day of household visit or during the previous seven days) was reported for two individuals (from two households) out of 18 individuals in the eight households that provided data on diarrhea. Using Poisson regression with cluster-robust variance estimation (to adjust for multiple individuals nested in households) [40], the likelihood of reported diarrhea was 25% higher for individuals living in homes where we detected *E. coli* and/or specific enteric pathogens (compared to households with no detection), though the association was not statistically significant (risk ratio [RR] = 1.25, cluster-robust standard error [SE] = 1.64, *p* = 0.865).

High blood pressure (hypertension) was reported for 31.6% (*n* = 6) out of 19 individuals living in five of the nine households. In addition to dietary exposure, sodium in drinking water is also associated with hypertension and cardiovascular disease [41,42,43]. In our study, sodium concentrations in source water samples ranged from 3.3–110.0 mg/L (mean = 66.0, SD = 36.0, median = 76.4, *n* = 9). The US EPA does not have a regulatory guideline value for sodium in drinking water, but recommends a sodium limit of 20 mg/L for individuals on sodium-restricted diets [44]. A recent World Health Organization (WHO) report noted that 71 of the 81 countries or territories globally that have drinking water standards for sodium use a value of 200 mg/L [45]. Therefore, to evaluate potential associations between sodium concentrations in source water samples and reported hypertension, we created a binary variable that equaled one if the sodium concentration was ≥100 mg/L (i.e., half the WHO reported value of 200 mg/L).

After adjusting for clustering by household, the likelihood of reported high blood pressure was 88% higher for individuals living in homes where we measured sodium concentrations in source water ≥ 100 mg/L (compared to households with sodium < 100 mg/L), though the association was not statistically significant (RR = 1.88, cluster-robust SE = 1.27, *p* = 0.353). After adjusting for reported tobacco use and age, the likelihood of reporting high blood pressure for those with sodium concentrations ≥ 100 mg/L increased to 400%; however, the association was again not significant (RR = 4.00, cluster-robust SE = 3.26, *p* = 0.088), and we did not have data to control for other known confounders, such as dietary sodium intake and obesity. There were no reports of current kidney or liver disease for any individuals (*n* = 19).

## 4. Discussion

In rural regions of many LMICs, as well as in many low-income rural (and urban) regions of the US, the United Nations Sustainable Development Goal of “…equitable access to safe and affordable drinking water for all” by 2030 remains out of reach [1,2]. Although limited secondary data indicate that many low-income regions of rural Appalachia lack sufficient access to safe drinking water [10,12], associated adverse impacts on health remain poorly understood. As far as we are aware, based on a recently completed systematic review [14], this study is one of <10 published drinking-water-focused research studies (based on primary data collection) conducted in Central Appalachia in the last ~20 years, and one of relatively few such studies conducted in the Appalachia region more broadly to measure bottled water quality and specific enteric pathogens.

In rural areas similar to the one reported on here, well water can be susceptible to fecal contamination from humans and animals via failing septic systems, runoff, and flooding. There is also some evidence that when pathogens are present in well water the home plumbing environment may contribute to further microbial growth, and, in turn, to higher pathogen concentrations in tap water samples [46,47]. Our finding that one third (33.3%, *n* = 3) of study households had enteric pathogens detected in their well water is concerning with regard to drinking-water-associated enteric disease exposure risk in this community. While PCR-based methods have the advantage of being able to detect viable, but non-culturable, bacteria, our detection of *E. coli* (via a culture-based detection method) in samples from only one of the three households where we detected specific pathogens suggests the use of *E. coli* as an indicator may underreport fecal contamination exposure risks in similar settings. 

Given the relatively high costs of bottled water for lower-income households, it is noteworthy that two thirds of study households (66.6%, *n* = 6) used bottled water as their primary drinking water source. Bottled water may offer protective advantages in rural settings with limited access to reliably safe drinking water sources. In our study, all three households with enteric pathogens detected in their well water reported using bottled water as their primary drinking water source. Our bottled water findings also parallel some observations from rural areas in LMICs. In particular, our observation that reported incomes were higher, and heads of household younger, overall, in households drinking primarily bottled water (compared with well water) aligns with findings from low-income households in rural areas of China [30,48].

Globally, bottled water use has grown rapidly over the last few decades, so much so that overall consumption in large LMICs now exceeds that of bottled water consumption in high-income countries, such as the US, by a substantial margin [49]. Long-term reliance on bottled water can be problematic for multiple reasons. While there are settings in which bottled water may be more cost effective than upgraded/advanced water treatment, typically, bottled water costs households much more than utility-supplied piped water. In addition, a lack of access to sufficient quantities of water is associated with adverse health outcomes [2,50,51]. Some low-income households that rely on bottled water may ration their use for drinking, and many households will use other, potentially unsafe, water sources for food preparation, cooking, and hygiene. Counterintuitively, for most US consumers, bottled water is not regulated under the SDWA, and, in the US and in LMICs, bottled water is not always as safe as consumers might expect [52,53,54]. US standards and testing frequencies are far more stringent for utility supplied water (EPA regulated) than for bottled water (FDA regulated), and, compared to water utilities, bottled water testing data are relatively difficult to access [55]. More broadly, the adverse environmental impacts from bottled water production, transport, disposal, and waste/pollution are considerable [56,57,58].

In the context of rural Appalachia and surrounding regions, drinking water and health-focused research studies based on relatively small sample sizes are not uncommon. For example, Pieper et al. studied well water, tap flushing, and lead concentration dynamics in 15 households in Virginia [59], Hunter et al. analyzed well water, septic systems, fecal indicator organisms, and antibiotics in 13 households in North Carolina [60], and Mulhern et al. analyzed activated carbon filter effectiveness and fecal indicator organisms in 17 households in North Carolina [61]. With respect to our study, while many of our results align with findings from similar research in LMIC settings, interpretation is limited by the small sample size (of both households and total individuals), as well as potential bias from our use of self-reported health outcomes, and a lack of data for homes that declined, or were unavailable, to participate. In addition, because we collected water samples during colder winter months, we cannot speak to potential seasonal impacts on water quality in this setting, an important factor considering studies in LMICs have documented increased rates of fecal contamination in water sources and diarrhea during warmer and wetter months [62,63], and because of the potential effects of seasonality on home plumbing distribution systems and associated impacts on tap water quality [64]. Consequently, it is unclear to what extent observations from this study may be generalizable to other rural areas of southwest Virginia or Central Appalachia.

## 5. Conclusions

Our findings contribute to the relatively limited research literature on drinking water and health in rural areas of Central Appalachia. Although our sample size was relatively small, our findings indicate that a considerable number of lower-income residents without utility-supplied water in rural southwest Virginia may be exposed to microbiological and/or chemical contamination in their water, and that many, if not most, rural households rely on bottled water as their primary source of drinking water. Our detection of specific enteric pathogens and relatively high concentrations of nitrate and iron in water samples indicates that additional research and data are needed to better understand which regions, communities, and populations in Central Appalachia may be exposed to contaminated water sources, the nature and extent of potentially associated adverse health outcomes, and what interventions might be implemented to expand safe water access.

## Figures and Tables

**Table 1:** Key Household Characteristics Overall and by Primary Source of Drinking Water.

	Private Well (*n* = 3)		Bottled Water (*n* = 6)		All Households (*n* = 9)	
	*n*	%	*n*	%	*n*	%
**HH Owned or Rented**						
Own	2	67%	4	80%	6	75%
Rent	1	33%	1	20%	2	25%
**Total** ^a^	**3**	**100%**	**5**	**100%**	**8**	**100%**
**Respondent’s Gender**						
Male	2	67%	4	67%	6	67%
Female	1	33%	2	33%	3	33%
**Total**	**3**	**100%**	**6**	**100%**	**9**	**100%**
**Respondent’s Race**						
White/Caucasian	3	100%	6	100%	9	100%
**HH Annual Income Level** ^b^						
<33 k	1	33%	1	17%	2	22%
33–38 k	1	33%	1	17%	2	22%
38–43 k	1	33%	1	17%	2	22%
43–48 k	0	0%	3	50%	3	33%
**Total**	**3**	**100%**	**6**	**100%**	**9**	**100%**
**Working toilet, sink, & tub/shower**						
Yes	3	100%	6	100%	9	100%
**Adults (≥18) residing in home**						
Total	4	n/a	11	n/a	15	n/a
**Children (<18) residing in home**						
Total	0	n/a	4	n/a	4	n/a
**Head of the HH Gender**						
Male	2	67%	4	67%	6	67%
Female	1	33%	2	33%	3	33%
**Total**	**3**	**100%**	**6**	**100%**	**9**	**100%**
**Head of HH: Age**						
Mean (standard deviation)	66.3	(7.5)	51.0	(17.1)	56.1	(16.0)
**Head of HH: Years lived in home**						
Mean (standard deviation)	30.7	(26.1)	12.6	(9.9)	18.6	(17.7)

Notes: HH = household, ^a^ missing data from one HH, ^b^ annual income is self-reported (thousands of US dollars per year). Shading provided to help delineate column-specific results.

**Table 2:** Water Sample Analysis Results by Water Source.

	Bottled Water Samples (*n* = 6)			Tap Water Samples (*n* = 9)			Source Water Samples ^a^ (*n* = 9)		
	Mean	SD	Max	Mean	SD	Max	Mean	SD	Max
**Physicochemical Parameters**									
pH	6.72	0.78	7.54	7.38	0.65	7.91	7.35	0.68	7.85
Temperature (Celsius)	16.4	5.2	22.1	16.3	3.4	20.9	13.3	2.0	15.6
Total dissolved solids (ppm) [Conductivity/2]	22.6	24.5	61.6	104.8	35.3	140.5	95.5	28.2	125.9
Dissolved Oxygen (%)	80.4	3.8	86.0	44.6	14.3	72.0	43.1	13.0	69.0
**Microbiological Indicators & Pathogens**									
Total Coliforms (TC) Detected: % HHs (*n*)	16.7%	(*n* = 1)		33.3%	(*n* = 3)		33.3%	(*n* = 3)	
MPN/100 mL for HHs with TC ^b^	1.0	n/a	2.0	5.5	7.3	18.3	5.1	8.0	16.9
*E. coli* (EC) Detected: % HHs (*n*)	0%	(*n* = 0)		11.1%	(*n* = 1)		11.1%	(*n* = 1)	
MPN/100 mL for HHs with EC ^b^	0.0	n/a	0.0	0.5	n/a	1.0	1.5	n/a	3.0
Specific Enteric Pathogens Detected	Not tested			Not tested			33.3%	(*n* = 3)	
*Aeromonas* bacteria: % HHs (*n*)							11.1%	(*n* = 1)	
*Campylobacter* bacteria: % HHs (*n*)							22.2%	(*n* = 2)	
*Enterobacter* bacteria: % HHs (*n*)							22.2%	(*n* = 2)	
Inorganic Chemicals with EPA MCL *									
Arsenic (ppb)	0.038	0.072	0.181	0.029	0.022	0.061	0.021	0.017	0.056
Barium (ppm)	0.005	0.008	0.021	0.137	0.120	0.272	0.135	0.119	0.274
Cadmium (ppb)	0.005	0.006	0.012	0.020	0.035	0.111	0.005	0.006	0.016
Chromium (ppm)	0.000	0.000	0.000	0.000	0.000	0.001	0.000	0.000	0.001
Copper (ppm)	0.001	0.003	0.008	0.066	0.183	0.552	0.002	0.003	0.009
Lead (ppb)	0.022	0.053	0.130	0.500	0.758	2.197	0.067	0.171	0.520
Nitrate [NO_3_^−^] (ppm)	4.575	1.580	6.300	4.278	3.261	10.400	4.533	1.551	7.000
Selenium (ppm)	0.000	0.000	0.000	0.000	0.000	0.000	0.000	0.000	0.000
**% HHs (*n*) with ≥1 parameter/s:**									
Greater than the EPA MCL ^c^	0%	(*n* = 0)		11.1%	(*n* = 1)		0%	(*n* = 0)	
Greater than ½ the EPA MCL ^c^	50.0%	(*n* = 3)		44.4%	(*n* = 4)		33.3%	(*n* = 3)	
**Chemicals with EPA SMCL**									
Aluminum (ppm)	0.006	0.012	0.030	0.004	0.003	0.010	0.002	0.001	0.003
Chloride (ppm)	2.583	3.854	10.048	9.346	7.399	23.706	9.308	7.331	23.524
Iron (ppm)	0.000	0.000	0.001	0.231	0.218	0.669	0.212	0.190	0.671
Manganese (ppm)	0.001	0.002	0.006	0.014	0.008	0.024	0.012	0.009	0.021
Sulfate [SO_4_] (ppm)	2.333	5.241	13.000	2.667	6.185	19.00	2.778	6.870	21.00
Zinc (ppm)	0.007	0.001	0.009	0.214	0.480	1.489	0.015	0.011	0.038
**% HHs (*n*) with ≥1 parameter/s:**									
Greater than the EPA SMCL ^d^	0%	(*n* = 0)		33.3%	(*n* = 3)		11.1%	(*n* = 1)	
Greater than ½ the EPA SMCL ^d^	0%	(*n* = 0)		55.5%	(*n* = 5)		66.6%	(*n* = 6)	

Notes: HH = household, ppm = parts per million (mg/L), ppb = parts per billion (μg/L). * Or EPA action levels and associated treatment techniques. ^a^ Source water = samples from tap after sterilizing and flushing (running) faucet for five minutes. ^b^ Max = maximum value of duplicate samples per source per HH. ^c^ Nitrate. ^d^ Iron. Shading provided to help delineate column-specific results.

## Data Availability

Upon reasonable request to the corresponding author, de-identified aggregated data may be provided.
